# Factors associated with self-report of polycystic ovary syndrome in the Coronary Artery Risk Development in Young Adults study (CARDIA)

**DOI:** 10.1186/s12905-023-02394-0

**Published:** 2023-05-09

**Authors:** Catherine Kim, Pamela J. Schreiner, David Siscovick, Ange Wang, Melissa F. Wellons, Imo Ebong, Thanh-Huyen Vu, Duke Appiah, Janet Catov, Enrique F. Schisterman, Zhe Yin, Cora E. Lewis

**Affiliations:** 1 Departments of Medicine, Obstetrics & Gynecology, and Epidemiology, University of Michigan, North Campus Research Center, 2800 Plymouth Road, Building 16, Room 405E, Ann Arbor, MI 48109 USA; 2 Division of Epidemiology and Community Health, University of Minnesota, Minneapolis, MN USA; 3 New York Academy of Medicine, New York City, NY USA; 4 Department of Obstetrics, Gynecology and Reproductive Sciences, University of California San Francisco, San Francisco, CA USA; 5 Department of Medicine, Vanderbilt University Medical Center, Nashville, TN USA; 6 Department of Medicine, University of California, Davis, Sacramento, CA USA; 7 Department of Preventive Medicine, Northwestern University, Chicago, IL USA; 8 Department of Public Health, School of Public and Population Health, Texas Tech University Health Sciences Center, Lubbock, TX USA; 9 Department of Obstetrics, Gynecology & Reproductive Sciences, University of Pittsburgh, Pittsburgh, PA USA; 10 Department of Biostatistics, Perelman School of Medicine, University of Pennsylvania, Philadelphia, PA USA; 11 Institute of Health Policy and Innovation, University of Michigan, Ann Arbor, MI USA; 12 Department of Epidemiology, University of Alabama at Birmingham, Birmingham, AL USA

**Keywords:** Polycystic ovary syndrome, Self-report, Diagnosis

## Abstract

**Background:**

Polycystic ovary syndrome (PCOS) is underdiagnosed, but factors associated with women’s report of diagnosis are not well-understood, particularly social determinants of health. Therefore, in a population-based cohort, we compared the characteristics of women with self-reported PCOS vs. women who have unrecognized PCOS vs. women without PCOS.

**Methods:**

We performed a secondary data analysis of the Coronary Artery Risk Development in Young Adults (CARDIA) Study, a population-based, prospective cohort of Black and White women. Participants were women (n = 2028) who responded to the question, “Did a doctor or nurse ever tell you that you had polycystic ovarian syndrome or polycystic ovarian disease?” at the year 15 examination. Women who answered “yes” were defined as having self-reported PCOS. Women who answered “no or not sure” were defined as having unrecognized PCOS if they also had irregular menses and hyperandrogenemia between 20 and 30 years of age. Exposures of interest included social determinants of health, symptoms including irregular menses and hirsutism, and comorbid conditions.

**Results:**

Forty-three (2.1%) of women had self-reported PCOS, 135 (6.7%) had unrecognized PCOS, and 1850 (91%) women were without PCOS. In logistic regression models adjusting for age, race, and center, women with self-reported PCOS were more likely to have obesity (OR 1.83, 95% CI 1.22, 2.75) and diabetes (OR 2.37, 95% CI 1.05, 5.33) compared to women without PCOS. Women with unrecognized PCOS were more likely to have hypertension (OR 1.68, 95% CI 1.03, 2.74) and food insecurity (OR 1.94, 95% CI 1.25, 3.01) compared to women without PCOS.

**Conclusions:**

Unrecognized PCOS is common. Self-report of PCOS is not associated with access to healthcare. Women who report PCOS are more often obese and comorbidities may contribute to recognition of PCOS.

**Supplementary Information:**

The online version contains supplementary material available at 10.1186/s12905-023-02394-0.

Polycystic ovary syndrome (PCOS), consisting of hyperandrogenism, ovulatory dysfunction, and polycystic ovaries, is a common endocrinopathy among reproductive-age women. [1, 2] Previous reports suggest that women with PCOS are frequently not diagnosed [3, 4] or experience delays in diagnosis. [5] In Australian cohorts, greater than two-thirds of women with PCOS did not report a PCOS diagnosis. [4, 6] Among primary care practices in the United Kingdom, [3] approximately half of women who met PCOS criteria did not have a PCOS diagnosis.

It is not understood how women who report having PCOS differ from women who do not report PCOS but nevertheless meet criteria. Several factors could potentially contribute to women’s self-report of PCOS. First, previous reports suggest that women with PCOS have poorer social determinants of health (SDoH) than women without PCOS. [7, 8] Thus, some women may not be diagnosed due to barriers to medical services. Second, few reports of PCOS include substantial numbers of Black women. Existing reports note the higher prevalence of obesity and insulin resistance among Black women with PCOS compared to White women with PCOS [9–11], although it is unclear if this reflects the patient populations in electronic health records or referral centers as opposed to population-based studies. Third, cardiometabolic abnormalities are sometimes but not always present among women with PCOS, [12, 13] and it is possible that women with lesser comorbidity are recognized less frequently than women with obesity and diabetes. Fourth, several PCOS phenotypes exist, [14] and the heterogeneity of definitions may contribute to diagnostic uncertainty. This uncertainty may be exacerbated due to differing guidelines for assessing and defining hyperandrogenemia, [15] ovulatory dysfunction, [15] and polycystic ovarian morphology. [16] PCOS is sometimes defined as the presence of any two of these three criteria, [17] although at least one guideline requires that hyperandrogenemia be present. [18].

Enhancing PCOS recognition by women and their providers may eventually reduce associated morbidities associated with PCOS, including subfertility, depressive disorders, and cardiovascular risk. Therefore, we examined factors associated with women’s self-report of PCOS using data from the Coronary Artery Risk Development in Young Adults (CARDIA) study, [19] a population-based observational cohort which included Black and White women. We hypothesized that greater salience of the diagnosis, including symptoms of hyperandrogenemia and cardiometabolic disorders, would be associated with increased self-report of PCOS. We also hypothesized that poorer SDoH profiles would be associated with unrecognized PCOS.

## Methods

### Study design

**Fig. 1:**
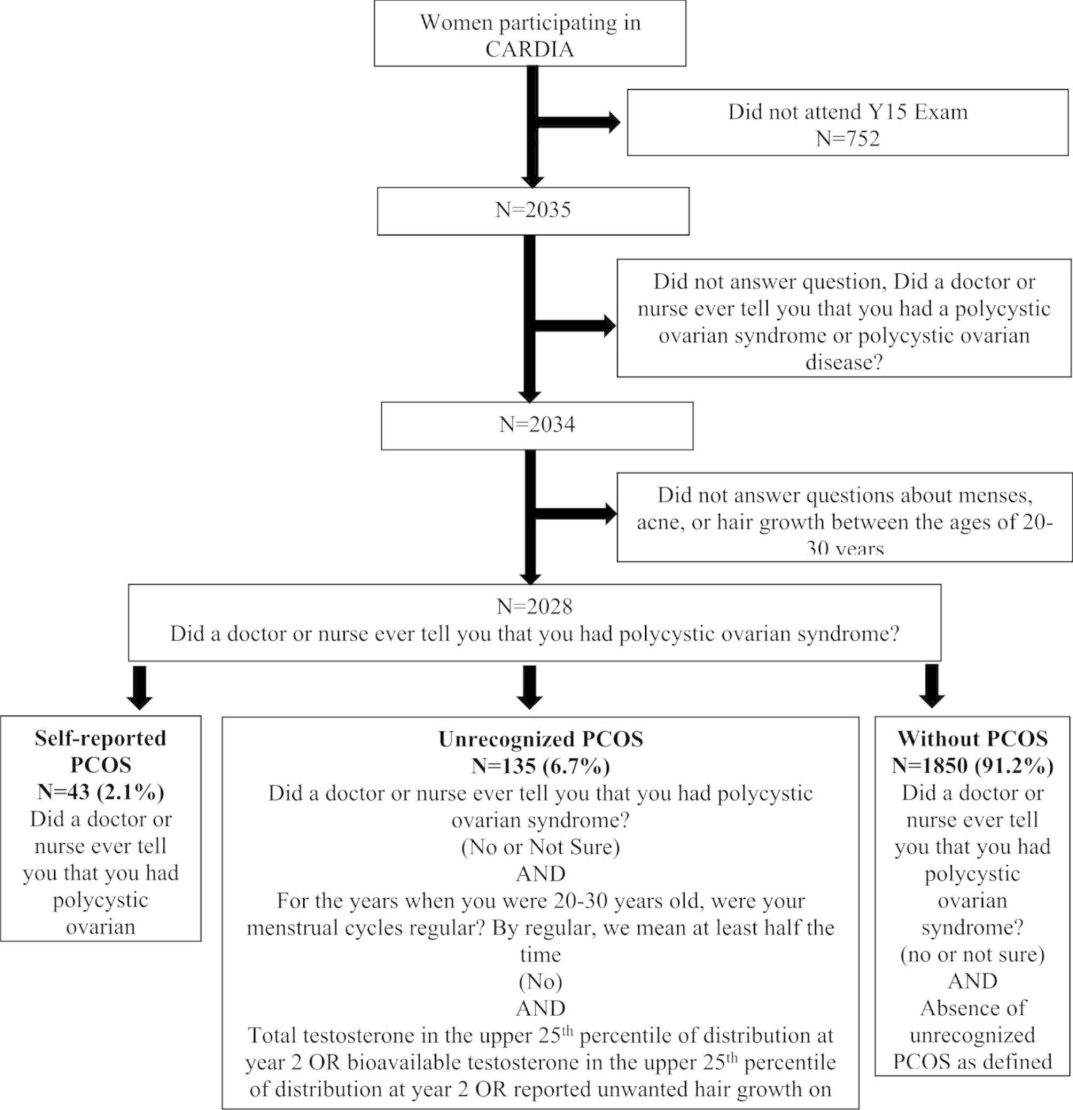
Flow chart of CARDIA participants and definitions of polycystic ovary syndrome (PCOS).

CARDIA is a multicenter, prospective, longitudinal cohort study of 5115 healthy Black and White adults from 4 US metropolitan populations (Birmingham, Alabama; Chicago, Illinois; Minneapolis, Minnesota; Oakland, California). [19] Participants were aged 18 to 30 years at baseline in 1985–1986 (year 0). Since enrollment, follow-up examinations were conducted in years 2, 5, 7, 10, 15 and every 5 years thereafter. Ethics approval was given by the institutional review boards from each field center and the coordinating center. Race was self-reported. Questions about the diagnosis of PCOS were included at the Year 15 exam (Y15), which was attended by 73% of surviving women (Fig. 1). Seven women did not respond to questions enquiring about the diagnosis of PCOS, menses, or hair growth, leaving a total of 2028 female participants available for analysis.

### Self-reported and unrecognized PCOS

Figure 1 shows how women were classified into the mutually exclusive categories of self-reported PCOS, unrecognized PCOS, and women without PCOS. At Y15, when women were aged 33–45, they were asked, “Did a doctor or nurse ever tell you that you had polycystic ovarian syndrome or polycystic ovarian disease?” Women who responded “yes” were classified as having self-reported PCOS. These women were also asked about age at diagnosis and what therapies they had received.

Women were classified as having unrecognized PCOS if they responded, “no or not sure” to “Did a doctor or nurse ever tell you that you had polycystic ovarian syndrome or polycystic ovarian disease?” and also had evidence of both ovulatory dysfunction and hyperandrogenism. Ovulatory dysfunction was defined by self-reported menstrual irregularity; at the Y15 exam, women were asked, “For the years when you were 20–30 years old, were your menstrual cycles regular? By regular, we mean at least half the time.” Women who responded “no” were defined as having irregular menses. Identification of hyperandrogenism was based on either a self-report measure of hirsutism or circulating androgen levels. For our measure of hirsutism, women were asked, “When you were 20–30 years old, did you ever have unwanted hair growth on your face, back, chest, arms, thighs, or legs? Do not include hair growth in the lower leg or underarm area.” Women who answered “yes” were defined as having symptoms of hyperandrogenemia.

Through the ancillary CARDIA Women’s Study (CWS), 1370 of the 2028 participants (68%), testosterone and sex hormone binding globulin (SHBG) were measured from samples collected at the Year 2 exam. When analyses were restricted to only women with circulating androgen measures, the results were similar to those from analysis including the self-report measure of hirsutism (Additional File Table 1). Therefore, analyses including both women with and without biochemical androgen measures are presented.

Total testosterone and SHBG measurements were performed by the OB/GYN Research and Diagnostic Laboratory at the University of Alabama, Birmingham. Testosterone was measured using a competitive immunoassay (Beckman Coulter, Fullerton, CA) using direct chemiluminescent technology on the Beckman Access Automated System. Free testosterone was calculated on the basis of measured total testosterone and SHBG. [20] Based on the 75th percentile of values at year 2, biochemical hyperandrogenism was defined as > 52 ng/dL total T or 0.37 ng/dL free testosterone. Of the 2028 participants, 781 (n = 39%) had hyperandrogenemia by report of hirsutism between the ages of 20–30 and/or elevations in total or free testosterone. Hirsutism and elevated androgens identified overlapping but different populations of women (Additional File Table 2): women with both hirsutism and elevations in androgens were the most likely to report acne, infertility, and obesity.

### Covariates

In addition to ascertainment of irregular menses and hirsutism at the Y15 exam, women were asked, “When you were 20–30 years old, did you ever have acne?” Women were also asked, at Y15, if they used oral contraceptive pills (OCPs) between 20 and 30 years of age. Responses to this question had high agreement with women’s responses to current OCP use at the Year 2 exam when women were approximately 27 years of age, with only 0.9% of discordance between report of current use of OCPs at Year 2 exam with recall of OCP use between 20 and 30 years of age at Y15. At Y15, women were asked about SDoH, including education, income, food insecurity, difficulty paying for basic needs, and access to medical care. The subset of CWS participants were also asked, “Have you and a male partner ever had unprotected sexual intercourse for at least 12 months without becoming pregnant?” Women who responded “yes” were classified as having infertility.

At Y15, body weight was measured to the nearest 0.2 kg with a calibrated, balance-beam scale. Height was measured with a vertical ruler to the nearest 0.5 cm. Body mass index (BMI) was calculated as weight in kilograms divided by height in meters squared, and obesity was defined as BMI ≥ 30 kg/m^2^. After 5 min of rest, blood pressure was measured from participants in the seated position 3 times at 1-minute intervals; the average of the last 2 measurements was used. Hypertension was defined according to the criteria of the Joint National Committee 6 which were current at Y15: systolic blood pressure ≥ 140 mm Hg, diastolic blood pressure ≥ 90 mm Hg, or current use of antihypertensive medication. [21] Diabetes was defined as a fasting glucose level of at least 126 mg/dL at examinations 0, 7, 10, or 15 or the self-reported use of oral hypoglycemic medications or insulin at any examination. [22] Plasma concentrations of total cholesterol, high-density lipoprotein cholesterol, and triglycerides were measured at all examinations using enzymatic methods at Northwest Lipids Research Laboratory. Hypercholesterolemia was defined as low-density lipoprotein estimated by Friedewald equation > 160 mg/dL at any follow-up examination or use of cholesterol-lowering medication. [22].

### Statistical analysis

Participant characteristics by category of self-reported PCOS, unrecognized PCOS, and no PCOS were defined by means, medians, and proportions as appropriate (Table 1). Differences and trends between self-reported PCOS compared to without PCOS, and between unrecognized PCOS compared to without PCOS, were tested using t-tests, Wilcoxon tests, and χ^2^ analyses for continuous and categorical characteristics, respectively.

**Table 1:** Characteristics of women with self-reported PCOS, unrecognized PCOS, and no PCOS. Categories are mutually exclusive. N (%) or means (SD) shown

	Self-reported PCOS	Unrecognized PCOS	No PCOS	p-value, self-reported PCOS v. No PCOS	p-value, unrecognized PCOS v. No PCOS
	N = 43	N = 135	N = 1850		
Age at year 15 exam (years)	40.8 (3.8)	39.8 (4.0)	40.2 (3.7)	0.25	0.28
Black (n, %)	19 (44%)	66 (49%)	922 (50%)	0.46	0.83
Previous pregnancy (n, %)	34 (79%)	113 (84%)	1527 (83%)	0.75	0.77
Infertility (n, %)^*^	14 (33%)	42 (31%)	369 (20%)	**0.042**	**0.002**
Hirsutism between 20–30 years (n, %)†	20 (47%)	88 (65%)	361 (20%)	**< 0.001**	**< 0.001**
Acne between 20–30 years (n, %)	18 (42%)	70 (52%)	783 (42%)	0.95	**0.03**
OCP use between 20–30 years (n, %)	29 (67%)	92 (68%)	1393 (75%)	0.24	0.06
Irregular menses between 20–30 years, (n, %)†	15 (35%)	135 (100%)	327 (18%)	**< 0.001**	**< 0.001**
Total testosterone (ng/dl) ^*^ ‡	44 (22, 70)	56 (39, 69)	37 (24, 50)	0.15	**< 0.001**
Free testosterone (ng/dl) ^*^ ‡	0.25 (0.17, 0.49)	0.42 (0.26, 0.55)	0.22 (0.13, 0.34)	0.07	**< 0.001**
BMI at year 2 (kg/m^2^)	28.3 (6.79)	26.13 (6.68)	25.08 (5.94)	**< 0.001**	0.052
BMI at year 15 (kg/m^2^)	32.2 (7.9)	29.4 (8.6)	29.0 (7.6)	**0.0065**	0.52
BMI category at year 15 (n, %)				**0.018**	0.30
<25 kg/m^2^	8 (19%)	50 (38%)	670 (37%)		
25-29.9 kg/m^2^	11 (26%)	27 (21%)	482 (27%)		
≥30 kg/m^2^	24 (56%)	54 (41%)	666 (37%)		
Hypertension at year 15 (n, %)	6 (14%)	28 (21%)	263 (14%)	0.96	**0.039**
Diabetes at year 15 (n, %)	8 (18%)	14 (10%)	146 (8%)	**0.02**	0.31
Dyslipidemia at year 15 (n, %)	4 (9%)	6 (4%)	116 (6%)	0.35	0.39

^*^Available in the subset of participants in the ancillary CWS, n = 1163 for infertility and n = 1370 for androgen measures

†Unrecognized PCOS, by definition, consisted of hyperandrogenemia (by hirsutism or total testosterone elevations or free testosterone)

‡Median (interquartile range)

Polytomous logistic regression was conducted to assess the associations between the outcome of PCOS category and independent variables grouped into symptoms (Table 2, Model 1), SDoH (Table 2, Model 2), comorbidities (Table 2, Model 3), and all of these factors together (Table 2, Model 4). In these models, separate odds ratios (ORs) were generated for the PCOS groups with women without PCOS as a referent. Symptom and comorbidity variables were selected for inclusion in multivariable models based upon significant associations with PCOs category in unadjusted comparisons shown in Table 1. SDoH variables were selected for inclusion in multivariable models based upon significant associations with PCOS category in unadjusted comparisons shown in Table 3. All models in Table 2 adjusted for age, race, and center.

**Table 2:** Association between symptoms of hyperandrogenemia and ovulatory dysfunction, social determinants of health, and comorbidities with the outcome of PCOS category, defined as self-reported PCOS, unrecognized PCOS, or no PCOS. Odds ratios and 95% confidence intervals (OR, 95% CI) shown. All models adjust for age, race (Black vs. White), and field center

	Self-reported PCOS, Reference = no PCOS OR (95% CI)	Unrecognized PCOS, Reference = no PCOS OR (95% CI)	
Model 1: Symptoms of hyperandrogenism and ovulatory dysfunction			
Unwanted hair growth during 20-30 s	**4.58 (2.41, 8.7)**	**17.22 (10.1, 29.35)**	
Acne during 20-30 s	0.85 (0.45, 1.59)	1.3 (0.79, 2.15)	
Irregular menses during 20-30 s	**3.70 (1.85, 7.14)**	*	
OCP use during 20 – 30 s	0.63 (0.32, 1.24)	**0.24 (0.13, 0.44)**	
Model 2: Social determinants of health			
Food insecurity	0.93 (0.37, 2.36)	**1.94 (1.25, 3.01)**	
Did not seek care because of cost or lack of coverage	0.97 (0.34, 2.75)	1.2 (0.69, 2.10)	
Very hard, fairly hard, not too hard to get health services	1.75 (0.86, 3.56)	1.31 (0.86, 2.01)	
Model 3: Comorbidities			
BMI category at year 15	**1.83 (1.22, 2.75)**	1.04 (0.83, 1.3)	
Hypertension at year 15	0.75 (0.3, 1.91)	**1.68 (1.03, 2.74)**	
Diabetes at year 15	**2.37 (1.05, 5.33)**	1.26 (0.7, 2.29)	
Model 4: Symptoms, social determinants of health, and comorbidities			
Unwanted hair growth during 20-30 s	**4.11 (2.13, 7.91)**	**16.8 (9.72, 29.06)**	
Acne during 20-30 s	0.87 (0.46, 1.66)	1.38 (0.82, 2.31)	
Irregular menses during 20-30 s	**3.70 (1.85, 7.69)**	^*^	
OCP use during 20-30 s	0.63 (0.32, 1.25)	**0.25 (0.13, 0.46)**	
Food insecurity	0.77 (0.32, 1.88)	1.73 (0.87, 3.44)	
Did not seek care because of cost or lack of coverage	1.14 (0.45, 2.93)	1.38 (0.60, 3.17)	
Very hard, fairly hard, not too hard to get health services	1.46 (0.71, 2.98)	0.91 (0.49, 1.7)	
BMI category at year 15	**1.71 (1.13, 2.59)**	1.17 (0.86, 1.60)	
Hypertension at year 15	0.76 (0.29, 1.94)	1.60 (0.77, 3.33)	
Diabetes at year 15	2.18 (0.95, 5.04)	0.86 (0.37, 1.96)	

*By definition, the odds of having irregular menses are incorporated into the definition of unrecognized PCOS, and inclusion of this variable led to unstable model assumptions.

**Table 3:** Social determinants of health among women with self-reported PCOS, unrecognized PCOS, and no PCOS. Categories are mutually exclusive. N (%) or means (SD) shown

	Self-reported PCOS	Unrecognized PCOS	No PCOS	p-value, self-reported PCOS v. no PCOS	p-value, unrecognized PCOS v. no PCOS
	N = 43	N = 135	N = 1850		
High school education or less (n, %)	5 (12%)	30 (22%)	389 (21%)	0.13	0.74
Household income < $50,000 (n, %)	16 (37%)	64 (47%)	798 (43%)	0.44	0.33
Married (n, %)	19 (44%)	69 (51%)	944 (51%)	0.38	0.98
Difficulty paying for basics (n, %)	10 (23%)	37 (27%)	411 (22%)	0.87	0.16
Food insecurity (n, %)	7 (16%)	36 (27%)	291 (16%)	0.92	**< 0.001**
Usual source of medical care (n, %)	41 (95%)	132 (98%)	1785 (96%)	0.69	0.43
Type of usual medical care (n, %)				0.69	0.33
Private physician/HMO	38 (88%)	112 (83%)	1537 (83%)		
Walk in clinic	1 (2.3%)	7 (5.2%)	79 (4.3%)		
Other clinic	1 (2.3%)	8 (5.9%)	126 (6.8%)		
Emergency department	1 (2.33)	5 (3.70)	29 (1.57)		
Private insurance or HMO (n, %)	39 (90%)	116 (86%)	1567 (85%)	0.28	0.70
Medicaid or Medicare (n, %)	1 (2%)	14 (10%)	116 (6%)	0.52	0.06
Out of pocket cost (n, %)	32 (74%)	112 (83%)	1504 (81%)	0.25	0.63
Distance from home to usual source of medical care (minutes)	15 (10)	19 (12)	18 (14)	0.15	0.26
Have not always had health insurance in the past 2 years (n, %)	40 (93%)	114 (84%)	1649 (89%)	0.62	0.095
Did not seek care because of cost or lack of coverage (n, %)	7 (16%)	23 (17%)	210 (11%)	0.33	**0.048**
Very hard, fairly hard, not too hard to get health services (n, %)	17 (40%)	50 (37%)	518 (28%)	0.097	**0.02**
Very hard, hard, somewhat hard to pay for medical care (n, %)	10 (23%)	31 (23%)	352 (19%)	0.49	0.26

We examined interactions of the independent variables with race to determine whether patterns of associations differed between Black and White women as noted in previous reports. [9–11] In sensitivity analyses, we constructed models without race to determine if this changed associations between SDoH and PCOS category. We also examined whether the pattern of associations changed when BMI at Y2, rather than Y15, was examined as a predictor. We examined whether the results changed when PCOS was defined using androgen levels at the upper 5th rather than upper 25th percentile. Finally, we compared women with unrecognized and self-reported PCOS in polytomous logistic models that used unrecognized PCOS as the reference group. Analyses were performed using SAS version 9.4 (SAS Institute Inc, https://support.sas.com/software/94/).

## Results

Table 1 shows the characteristics of women with self-reported PCOS (2.1%), unrecognized PCOS (6.7%), and without PCOS (91%). Across these categories, women had similar age and race distributions. Women with self-reported PCOS reported their age at diagnosis as 32.5 (SD 8.1) years; 10 (23%) reported undergoing ovarian surgery, 6 (14%) reported having taken medications for hirsutism or acne, 17 (40%) reported having taken medications for irregular menses, 9 (21%) reported having taken medications for infertility. Only 15 (35%) also had hyperandrogenemia and irregular menses.

In unadjusted comparisons (Table 1), women with self-reported PCOS were significantly more likely than women without PCOS to have had hirsutism, histories of irregular menses, infertility, obesity, and diabetes. Women with unrecognized PCOS were similar to women without PCOS regarding obesity and diabetes, but the former were more likely to have hypertension and elevated testosterone levels than the latter. Women with self-reported PCOS and women without PCOS had similar SDoH (Table 3). Women with unrecognized PCOS were significantly more likely than women without PCOS to report food insecurity, not seeking medical care because of cost or lack of coverage, and difficulty getting health services.

Table 2 shows how women with self-reported PCOS differed from women without PCOS after adjustment for age, race, and center. Compared to women without PCOS, women with self-reported PCOS were still more likely to have hirsutism and irregular menses (Model 1), as well as obesity and diabetes (Model 3). However, women with self-reported PCOS had similar SDoH as women without PCOS (Model 2). When symptoms, SDoH, and comorbidities were all included in Model 4, hirsutism, irregular menses, and obesity were still associated with self-reported PCOS.

Table 2 also shows how women with unrecognized PCOS differed from women without PCOS after adjustment for age, race, and center. Women with unrecognized PCOS were more likely to have hirsutism (Model 1), to report food insecurity (Model 2), and to have hypertension (Model 3), and less likely to report OCP use (Model 1). When symptoms, SDoH, and comorbidities were all included in Model 4, hirsutism and lack of OCP use between 20 and 30 years of age were the only factors associated with unrecognized PCOS.

Interactions by race were not significant at p < 0.10, so results are shown with adjustment for race rather than stratification by race. In sensitivity analyses, models that did not adjust race found similar patterns of associations between SDoH and PCOS category (Additional File Table 3). When women who answered “not sure” regarding a diagnosis of PCOS were excluded, the pattern of associations were similar. Examination of BMI at Y2 rather than Y15 yielded a similar pattern of results: women with self-reported PCOS had higher odds of having obesity at Y2 compared to women without PCOS (OR 1.46, 95% CI 0.98, 2.16). When we redefined abnormal androgen levels of total and free testosterone in the upper 5th percentile, rather than the upper 25th percentile, the prevalence of unrecognized PCOS dropped to 4.8% (n = 97); however, the pattern of results based on this definition was similar to the primary analysis (Additional File Table 4), except that women with unrecognized PCOS were slightly more likely to report difficulty accessing healthcare than women without PCOS, and the association between hypertension and unrecognized PCOS was no longer significant. When we compared women with self-reported vs. unrecognized PCOS in a polytomous model that had unrecognized PCOS as the reference group (Additional File Table 5), women with self-reported PCOS were less likely to report unwanted hair growth, more likely to use OCPs, and were more likely to be obese but did not differ in SDoH. Finally, due to the conduction of multiple comparisons in adjusted models, we examined the significance of the associations with PCOS category in Table 2 using the Bonferroni correction. [23] Associations between hirsutism, irregular menses, OCP use, and BMI remained significant, but associations with food insecurity and hypertension were no longer significant. In the combined Model 4, the association with BMI was no longer significant (p = 0.031).

## Discussion

PCOS may affect as many as 1 out of 10 women, [24] but the frequency of under-recognition and the factors contributing to self-report are poorly understood. In this population-based sample of Black and White women, unrecognized PCOS was common. Compared to women without PCOS, women with self-reported PCOS were more likely to have obesity and diabetes, as well as hyperandrogenemia and ovulatory dysfunction. Women with unrecognized PCOS had similar likelihood of obesity and diabetes compared to women without PCOS. After adjustment for age and race, we did not find that women with unrecognized PCOS had marked differences in SDoH compared to women without PCOS, except women with unrecognized PCOS did report greater food insecurity.

In our study, approximately 2% of women had self-reported PCOS, similar to the prevalence reported in a large United Kingdom primary care database, [3] private insurance claims data in the United States, [25] an integrated health care delivery system in northern California, [11] and self-report in Australia. [4] This prevalence is lower than that reported for smaller studies that systematically screened for PCOS, which estimate prevalence at approximately 10% depending upon the diagnostic scheme used. [4, 26, 27] In one of these Australian studies, March and colleagues noted that 68% of women who met diagnostic criteria for PCOS as defined by hyperandrogenism and irregular menses did not carry a PCOS diagnosis, and 69% of women who met diagnostic criteria for PCOS as defined by polycystic ovaries and irregular menses or hyperandrogenism did not carry a PCOS diagnosis. [4] This discrepancy is similar to what we noted, suggesting that the majority of women who meet criteria for PCOS remain unrecognized even by 40 years of age, although it is possible that PCOS could be diagnosed later in life.

Our findings that self-reported PCOS was more strongly associated with obesity than unrecognized PCOS is aligned with previous reports noting that women identified with PCOS in subspecialty care were more likely to be obese than women identified with PCOS in employment screenings. [12] This likely reflects the greater morbidity of women referred to specialists for PCOS evaluation. [25] Our finding that women with unrecognized PCOS had higher odds of hypertension is similar to an Australian study, [28] although in our study, women with self-reported PCOS did not have higher odds of hypertension compared to women without PCOS, potentially due to the small numbers of women with hypertension and self-reported PCOS.

We did not find that SDoH varied by PCOS category, suggesting that these factors are not major factors in the recognition of PCOS. This may be due to associations between race and SDoH, but associations between SDoH and PCOS were similar whether or not we adjusted for race. The lack of association may also reflect the generally poor ascertainment of PCOS, regardless of healthcare setting. The exception is that women with unrecognized PCOS did report greater food insecurity than women without PCOS. A previous CARDIA study examined health behaviors among women who had PCOS defined by hyperandrogenism and irregular menses, but did not find differences in survey-assessments of energy intake, nutrients, dietary quality or physical activity with PCOS. [29] Thus, beyond the greater morbidity of women who self-reported PCOS, other explanations for the lack of self-report among women with PCOS remain largely unknown.

Strengths of this report include its population-based sample, inclusion of Black and White women, and inclusion of a significant proportion of women who were socioeconomically disadvantaged. Additional strengths include its assessment of comorbidities through examination rather than self-report as well as an extensive list of access to care factors. Limitations include the lack of confirmation of a women’s self-report of a diagnosis through medical record review. The CARDIA survey did not ask women who reported a health professional diagnosis about which criteria were used to diagnose them with PCOS, i.e., NIH criteria or Rotterdam criteria or Androgen Excess Society Criteria, nor did we ask which abnormalities (i.e. irregular menses, hirsutism, biochemical hyperandrogenemia, elevated antral follicle count) were used to identify them. Of note, this limitation regarding the heterogeneous nature of PCOS and grouping together of women meeting different criteria is also present in consortia that rely on electronic medical records, which include not only diagnoses by healthcare professionals but also specific symptom criteria for hirsutism, irregular menses, and polycystic ovaries. However, automated phenotyping using strict and broad classification criteria identified similar prevalence of PCOS, possibly due to shared genetic architecture. [9] Other limitations are that we classified hirsutism based upon self-report, rather than through examiner assessments of hirsutism using standardized scales. Ovarian imaging assessing presence of polycystic ovaries in the 3rd decade of life was not available, and so women who had PCOS based upon the presence of polycystic morphology used in the Rotterdam criteria were not captured in this report. Thus, our estimates of prevalence may be underestimates of true PCOS prevalence. We could not distinguish between use of OCPs for irregular menses, contraception, or both. Although PCOS is one of the most common endocrinopathies in reproductive-age women, only 2% of women reported having been told of a PCOS diagnosis; thus the power to detect significant associations, particularly after multivariable adjustment, was limited. The relatively small number of women with self-reported PCOS as compared to unrecognized PCOS limited exploration of race as an effect modifier. We performed multiple comparisons, and associations may have been detected by chance, although the significance of most associations persisted after conservative approaches to minimizing type I error in multiple comparisons. We did not have information on specialty care, including contacts with endocrinologists or gynecologists, which might be associated with higher odds of recognition.

## Conclusions

We conclude that women with self-reported PCOS may represent only a subset of women with PCOS. Despite their body mass and prevalence of diabetes similar to women without PCOS, women with unrecognized PCOS still had greater prevalence of infertility and hypertension compared to women without PCOS. Of note, we assessed access to care measures at Y15, and it is possible that examination of life course trajectories of SDoH could reveal potential differences by PCOS category. Larger population-based studies of factors associated with PCOS, particularly by phenotypes of PCOS, are needed to confirm these associations.

## Electronic supplementary material

Below is the link to the electronic supplementary material.

Additional File 1: Logistic regressionin women with hormonesonly

Additional File 2: Hirsutism and androgens

Additional File 3: Models without adjustment forrace

Additional File 4: Redefined hyperandrogenemia

Additional File 5: Unrecognized PCOS as referent

## Data Availability

The datasets used and/or analyzed during the current study are available from the corresponding author on reasonable request.

## List of Abbreviations

BMI: Body mass index
CARDIA: Coronary Artery Risk Development in Young Adults Study
CWS: CARDIA Women’s Study
OCPs: Oral contraceptive pills
PCOS: Polycystic ovary syndrome
SHBG: Sex hormone binding globulin
SDoH: Social determinants of health

## Declarations

### Attestation

Data regarding any of the subjects in the study has not been previously published unless specified. Data will be made available to the editors of the journal for review or query upon request.
