# Supplementary material for: Factors associated with self-report of polycystic ovary syndrome in the Coronary Artery Risk Development in Young Adults study (CARDIA)
Source: BMC Womens Health. 2023 May 9;23:248. doi: 10.1186/s12905-023-02394-0 (PMC10170674; doi:10.1186/s12905-023-02394-0)
Supplement: Supplementary file 1 — Additional File 1: Logistic regressionin women with hormonesonly [file 12905_2023_2394_MOESM1_ESM.docx]

| Supplemental Table 1. Association between symptoms, social determinants of health, and comorbidities with the self-report of PCOS, excluding women without any biochemical testosterone measures. Reference category is women without PCOS. All models adjust for age, race, and center. Odds ratios and 95% confidence intervals (OR, 95% CI) shown. | | |  |
| --- | --- | --- | --- |
|  | Recognized PCOS  OR (95% CI) | Unrecognized PCOS  OR (95% CI) |  |
| Model 1: Symptoms of hyperandrogenism and ovulatory dysfunction | | |  |
| Unwanted hair growth during 20s-30s | **2.79 (1.30, 5.99)** | **9.88 (5.33, 18.31)** |  |
| Acne during 20s-30s | 0.92 (0.44, 1.92) | 1.30 (0.74, 2.29) |  |
| Irregular menses during 20s-30s | **3.13 (1.37, 7.14)** | * |  |
| OCP use during 20s – 30s | 0.64 (0.29, 1.42) | **0.20 (0.09, 0.41)** |  |
|  |  |  |  |
| Model 2: Social determinants of health | | |  |
| Food insecurity | 0.7 (0.25, 1.98) | **1.77 (1.05, 2.98)** |  |
| Did not seek care because of cost or lack of coverage | 1.87 (0.7, 4.96) | 1.33 (0.71, 2.51) |  |
| Very hard, fairly hard, not too hard to get health services | 1.71 (0.76, 3.83) | 1.17 (0.71, 1.92) |  |
|  |  |  |  |
| Model 3: Comorbidities | | |  |
| BMI category at year 15 | **1.69 (1.04, 2.74)** | 1.01 (0.78, 1.31) |  |
| Hypertension at year 15 | 0.72 (0.26, 1.99) | 1.33 (0.75, 2.34) |  |
| Diabetes at year 15 | **2.97 (1.22, 7.27)** | 1.57 (0.82, 3.04) |  |
| *By definition, the odds of having irregular menses is incorporated into the definition of unrecognized PCOS, and inclusion of this variable led to unstable model assumptions. | | |  |
