# Supplementary material for: Factors associated with self-report of polycystic ovary syndrome in the Coronary Artery Risk Development in Young Adults study (CARDIA)
Source: BMC Womens Health. 2023 May 9;23:248. doi: 10.1186/s12905-023-02394-0 (PMC10170674; doi:10.1186/s12905-023-02394-0)
Supplement: Supplementary file 2 — Additional File 2: Hirsutism and androgens [file 12905_2023_2394_MOESM2_ESM.docx]

| Supplemental Table 2. Characteristics of women in sample with hirsutism only; elevated androgens only; and hirsutism AND elevated androgens. | | | | |
| --- | --- | --- | --- | --- |
|  | Hirsutism only,  without biochemical androgen elevations | Elevated androgens only, without hirsutism | Hirsutism and biochemical androgens elevations | p-value |
|  | N=322 | N=312 | N=147 |  |
| Age at year 15 exam (years) | 39.7 (3.8) | 40.3 (3.8) | 39.2 (3.7) | 0.008 |
| Black (n, %) | 178 (55%) | 143 (46%) | 80 (54%) | 0.04 |
| Previous pregnancy (n, %) | 267 (83%) | 259 (83%) | 110 (75%) | 0.07 |
| Acne between 20-30 years (n, %) | 172 (53%) | 130 (42%) | 89 (61%) | 0.0002 |
| OCP use between 20-30 years (n, %) | 238 (74%) | 206 (66%) | 90 (61%) | 0.012 |
| Irregular menses between 20-30 years**,** (n, %) | 92 (29%) | 66 (21%) | 37 (25%) | 0.097 |
| History of infertility (n, %) | 67 (42%) | 73 (33%) | 52 (48%) | 0.024 |
| Total testosterone (ng/dl) ^a^ | 31  (23, 39) | 58  (52, 69.5) | 60  (53, 76) | <0.0001 |
| Free testosterone (ng/dl) ^a^ | 0.19  (0.10, 0.25) | 0.43  (0.35, 0.54) | 0.47  (0.38, 0.67) | <0.0001 |
| BMI category at year 15 (n, %) |  |  |  | 0.013 |
| <25 kg/m^2^ | 107 (34%) | 99 (32%) | 32 (22%) |  |
| 25-29.9 kg/m^2^ | 75 (24%) | 85 (27%) | 32 (22%) |  |
| >30 kg/m^2^ | 132 (42%) | 127 (41%) | 83 (56%) |  |
| Hypertension at year 15 (n, %) | 55 (17%) | 59 (19%) | 28 (19%) | 0.80 |
| Diabetes at year 15 (n, %) | 23 (7%) | 32 (10%) | 13 (9%) | 0.38 |
| Dyslipidemia at year 15 (n, %) | 16 (5%) | 23 (7%) | 9 (6%) | 0.45 |
