# Supplementary material for: Factors associated with self-report of polycystic ovary syndrome in the Coronary Artery Risk Development in Young Adults study (CARDIA)
Source: BMC Womens Health. 2023 May 9;23:248. doi: 10.1186/s12905-023-02394-0 (PMC10170674; doi:10.1186/s12905-023-02394-0)
Supplement: Supplementary file 3 — Additional File 3: Models without adjustment forrace [file 12905_2023_2394_MOESM3_ESM.docx]

| Supplemental Table 3. Association between social determinants of health with the self-report of PCOS. Reference category is women without PCOS. All models adjust for age and center, but do not adjust for race. Odds ratios and 95% confidence intervals (OR, 95% CI) shown. | | |
| --- | --- | --- |
|  | Recognized PCOS  OR (95% CI) | Unrecognized PCOS  OR (95% CI) |
| Food insecurity | 0.88 (0.37, 2.07) | **1.76 (1.15, 2.69)** |
| Did not seek care because of cost or lack of coverage | 1.20 (0.48, 3.02) | 1.19 (0.69, 2.05) |
| Very hard, fairly hard, not too hard to get health services | 1.60 (0.80, 3.20) | 1.33 (0.87, 2.01) |
