# Supplementary material for: Factors associated with self-report of polycystic ovary syndrome in the Coronary Artery Risk Development in Young Adults study (CARDIA)
Source: BMC Womens Health. 2023 May 9;23:248. doi: 10.1186/s12905-023-02394-0 (PMC10170674; doi:10.1186/s12905-023-02394-0)
Supplement: Supplementary file 4 — Additional File 4: Redefined hyperandrogenemia [file 12905_2023_2394_MOESM4_ESM.docx]

| Supplemental Table 4. Sensitivity analysis, with hyperandrogenemia redefined as hirsutism or total testosterone in upper 5^th^ percentile or biochemical testosterone in the upper 5^th^ percentile. Association between symptoms, access to health variables, and comorbidities with the outcome of PCOS category, defined as self-reported PCOS, unrecognized PCOS, or without PCOS. Odds ratios and 95% confidence intervals (OR, 95% CI) shown. All models adjust for age, race (Black vs. White), and field center. | | |
| --- | --- | --- |
|  | Self-reported PCOS  OR (95% CI) | Unrecognized PCOS  OR (95% CI) |
| Model 1: Symptoms of hyperandrogenism and ovulatory dysfunction | | |
| Unwanted hair growth during 20s-30s | **4.75 (2.49, 9.04)** | **111.4 (48.0, 258)** |
| Acne during 20s-30s | 0.82 (0.43, 1.54) | 0.94 (0.47, 1.86) |
| Irregular menses during 20s-30s | **3.22 (1.64, 6.25)** | ^a^ |
| OCP use during 20s – 30s | 0.66 (0.34, 1.28) | **0.24 (0.1, 0.59)** |
|  |  |  |
| Model 2: Social determinants of health | | |
| Very hard, hard, or somewhat hard to pay for basics | 0.91 (0.42, 1.96) | 1.33 (0.82, 2.16) |
| Did not seek care because of cost or lack of coverage | 1.18 (0.47, 2.97) | 1.24 (0.67, 2.28) |
| Very hard, fairly hard, not too hard to get health services | 1.63 (0.81, 3.27) | **1.65 (1.02, 2.66)** |
|  |  |  |
| Model 3: Comorbidities | | |
| BMI category at year 15 | **1.83 (1.22, 2.75)** | 1.06 (0.82, 1.39) |
| Hypertension at year 15 | 0.74 (0.29, 1.88) | 1.65 (0.93, 2.91) |
| Diabetes at year 15 | **2.34 (1.04, 5.25)** | 1.09 (0.53, 2.25) |
