# Supplementary material for: Factors associated with self-report of polycystic ovary syndrome in the Coronary Artery Risk Development in Young Adults study (CARDIA)
Source: BMC Womens Health. 2023 May 9;23:248. doi: 10.1186/s12905-023-02394-0 (PMC10170674; doi:10.1186/s12905-023-02394-0)
Supplement: Supplementary file 5 — Additional File 5: Unrecognized PCOS as referent [file 12905_2023_2394_MOESM5_ESM.docx]

| Supplemental Table 5. Association between symptoms, access to health variables, and comorbidities, where unrecognized PCOS is the reference group. Odds ratios and 95% confidence intervals (OR, 95% CI) shown. All models adjust for age, race, and center. | |
| --- | --- |
|  | Self-reported PCOS  OR (95% CI) |
| Model 1: Symptoms of hyperandrogenism and ovulatory dysfunction | |
| Unwanted hair growth during 20s-30s | **0.27 (0.12, 0.58)** |
| Acne during 20s-30s | 0.65 (0.3, 1.4) |
| Irregular menses during 20s-30s | ^a^ |
| OCP use during 20s – 30s | **2.62 (1.1, 6.24)** |
|  |  |
| Model 2: Social determinants of health | |
| Very hard, hard, or somewhat hard to pay for basics | 0.77 (0.32, 1.83) |
| Did not seek care because of cost or lack of coverage | 0.93 (0.33, 2.65) |
| Very hard, fairly hard, not too hard to get health services | 1.15 (0.52, 2.56) |
|  |  |
| Model 3: Comorbidities | |
| BMI category at year 15 | **1.76 (1.11, 2.78)** |
| Hypertension at year 15 | 0.45 (0.16, 1.26) |
| Diabetes at year 15 | 1.87 (0.71, 4.98) |

^a^Irregular menses incorporated into the definition of unrecognized PCOS
